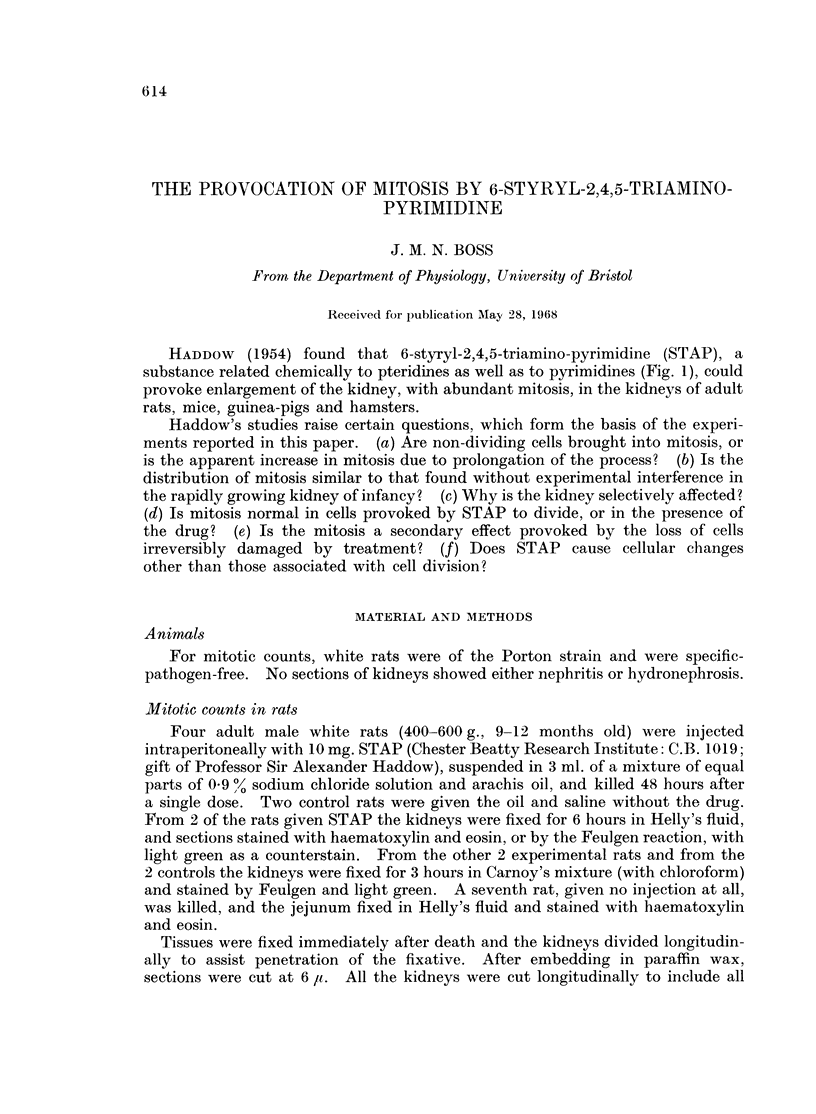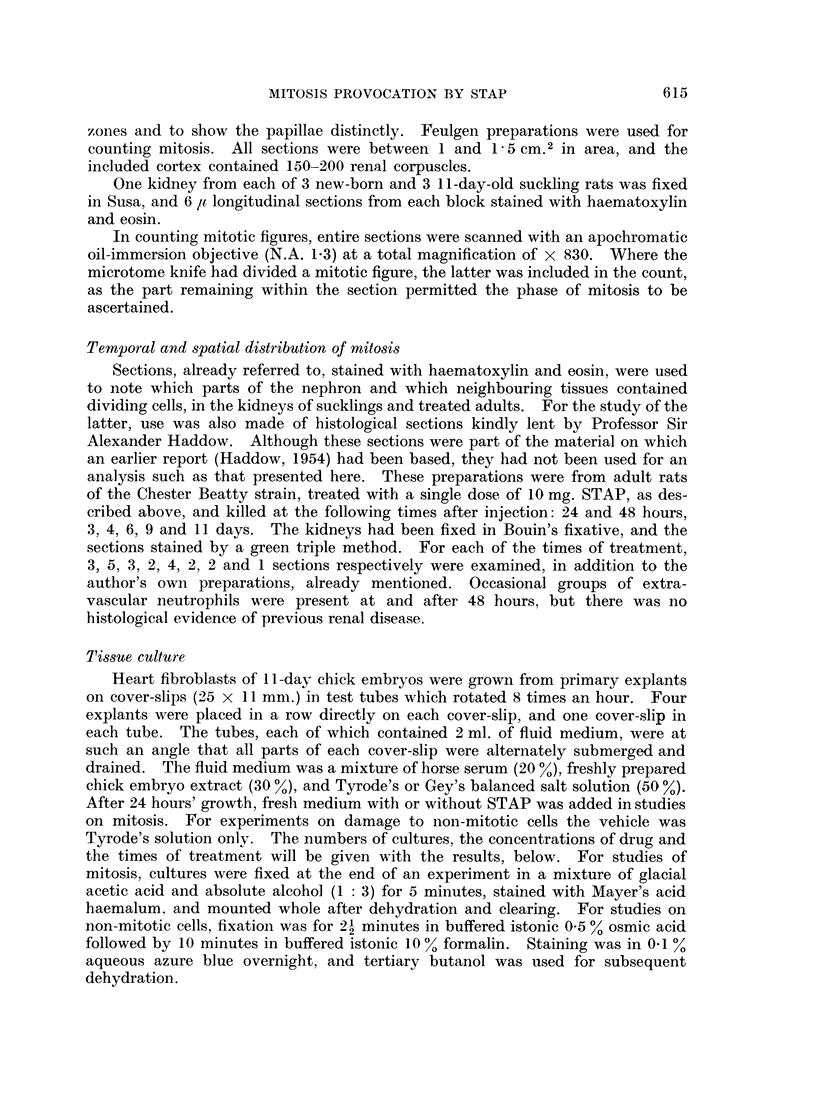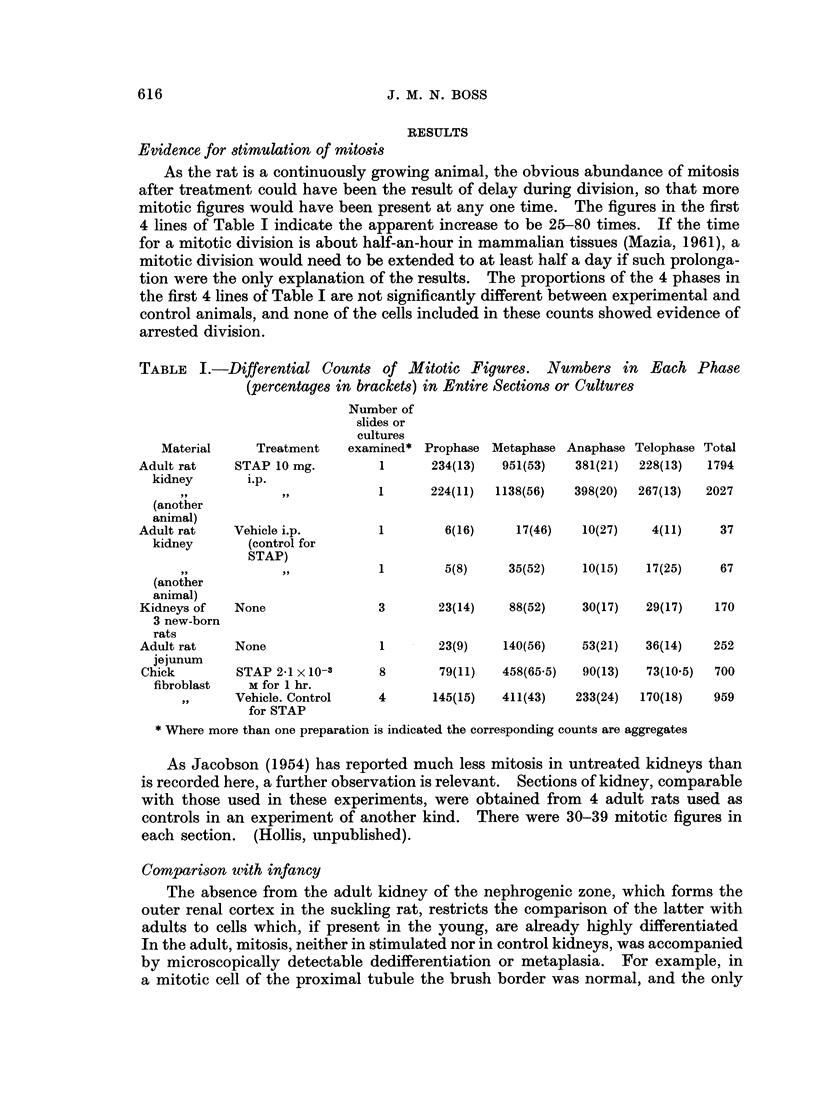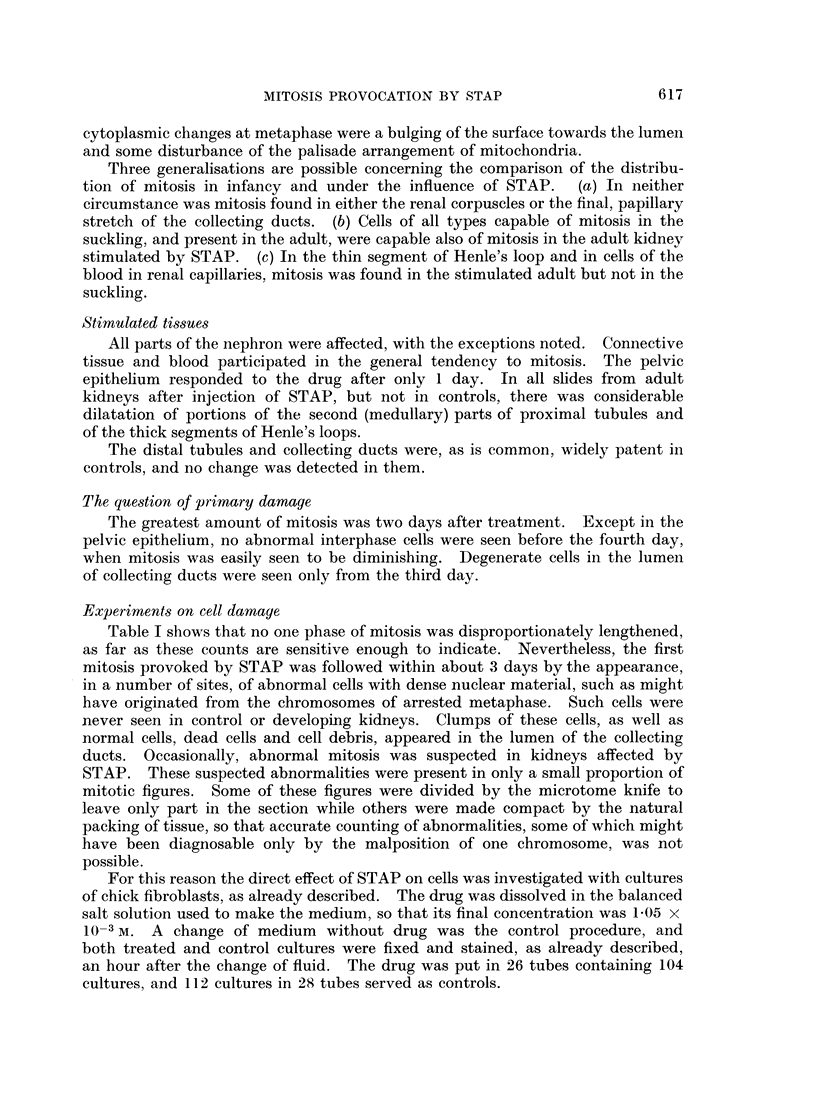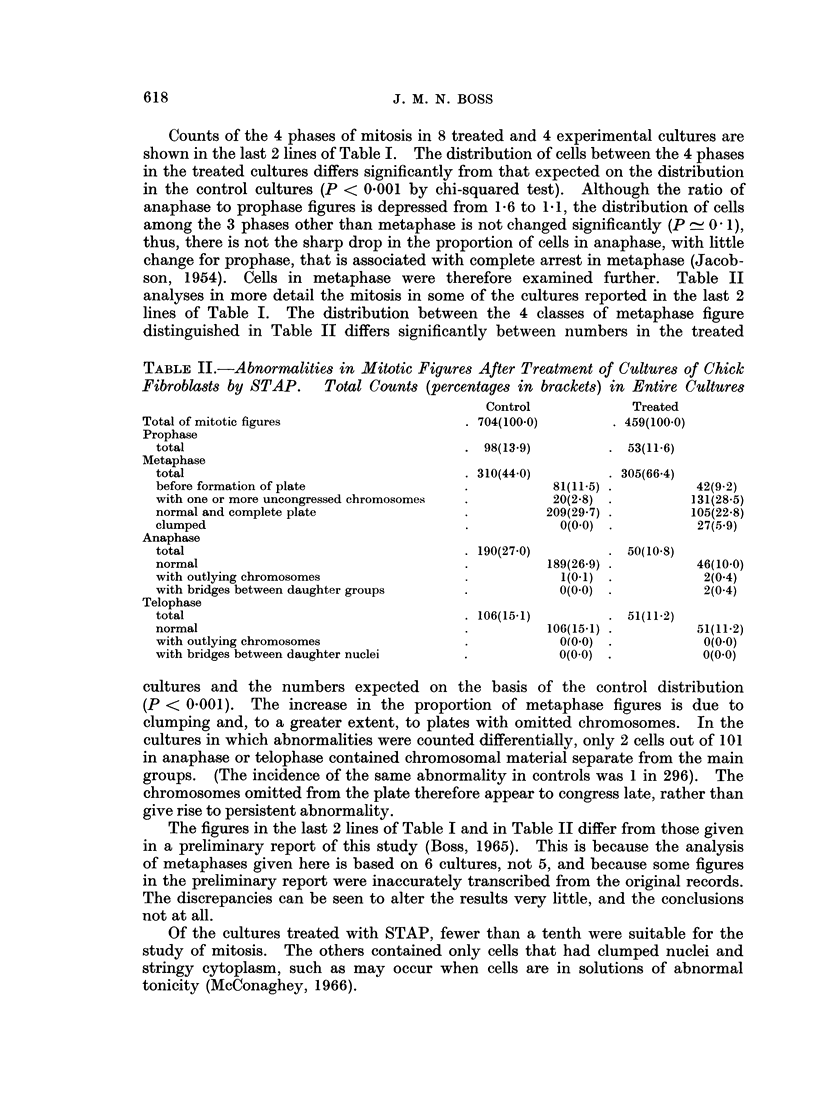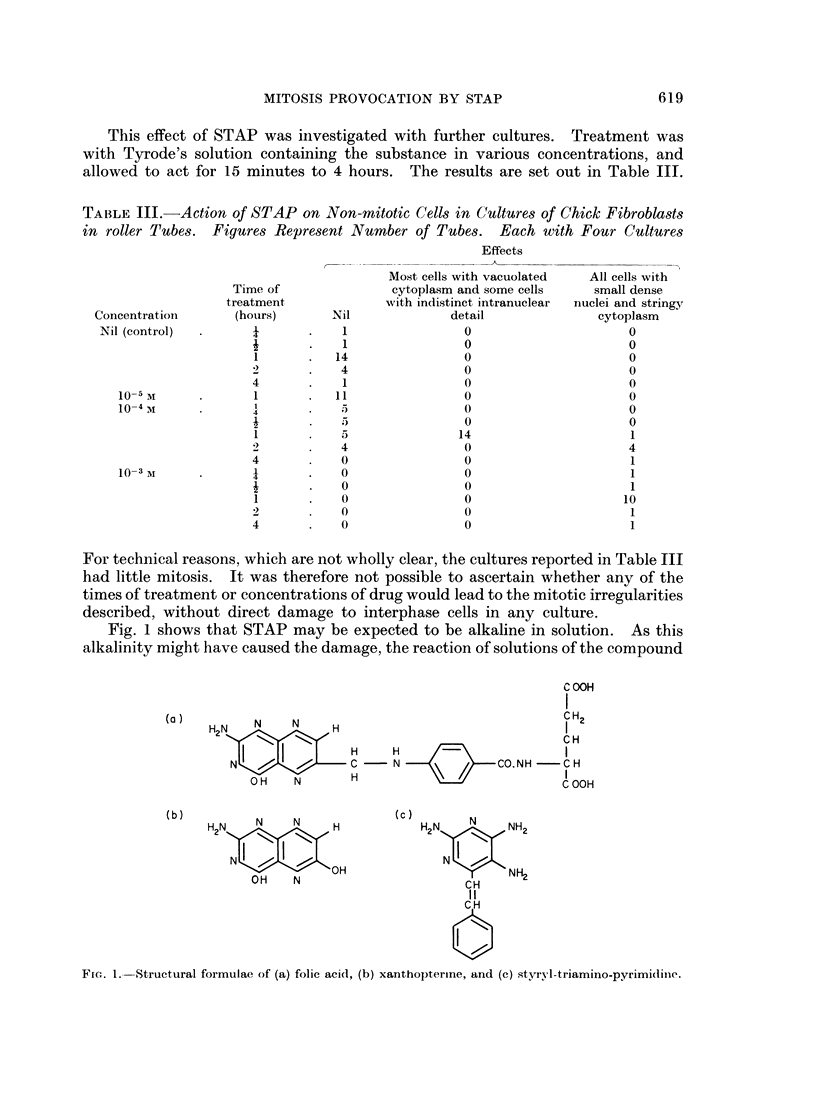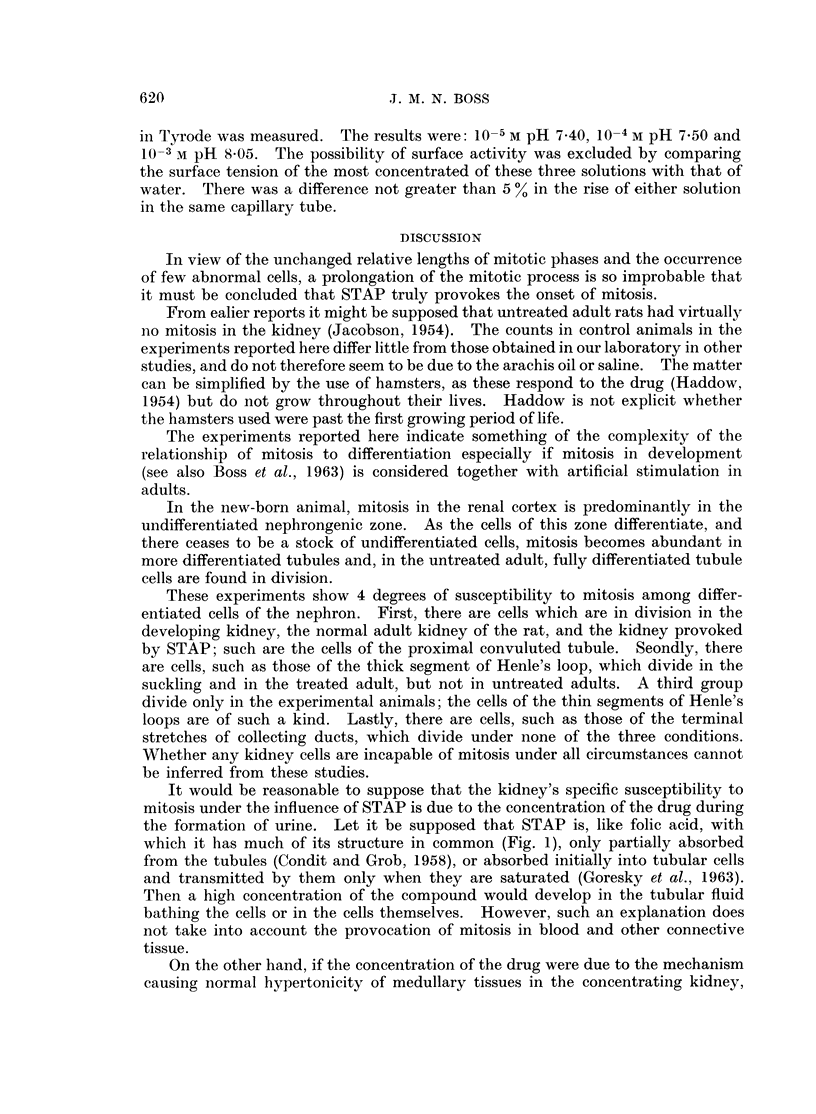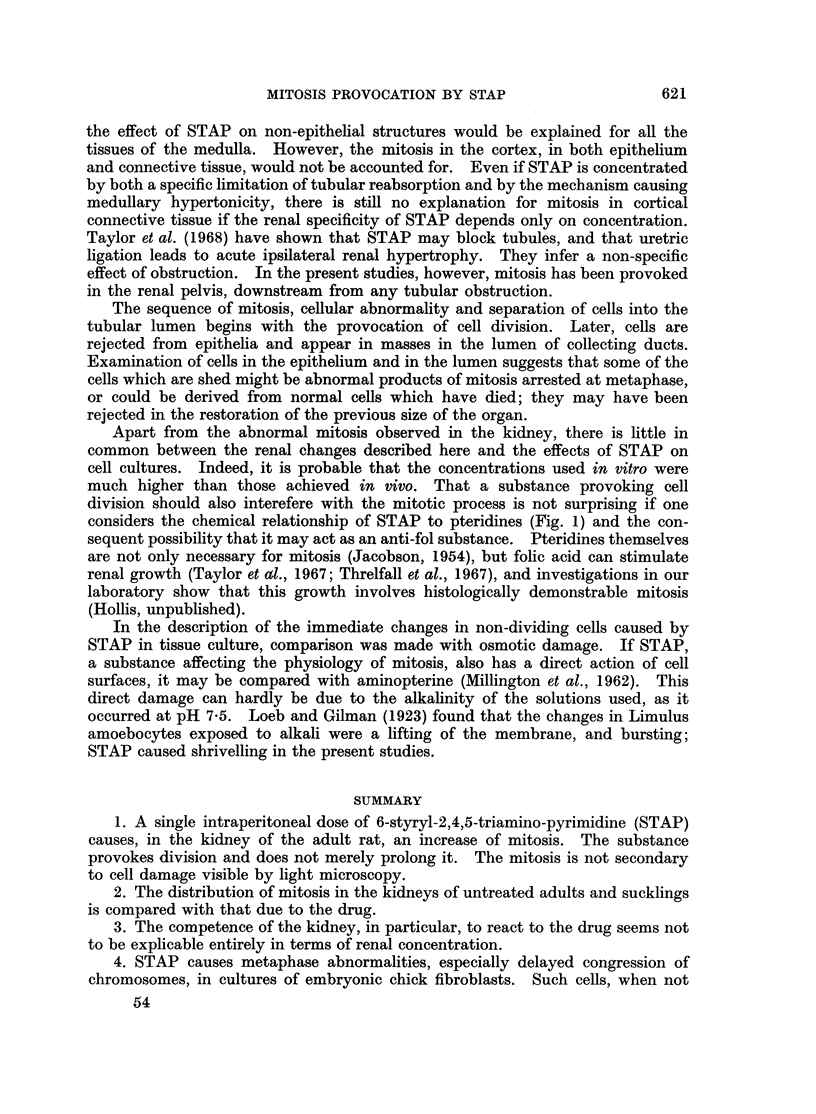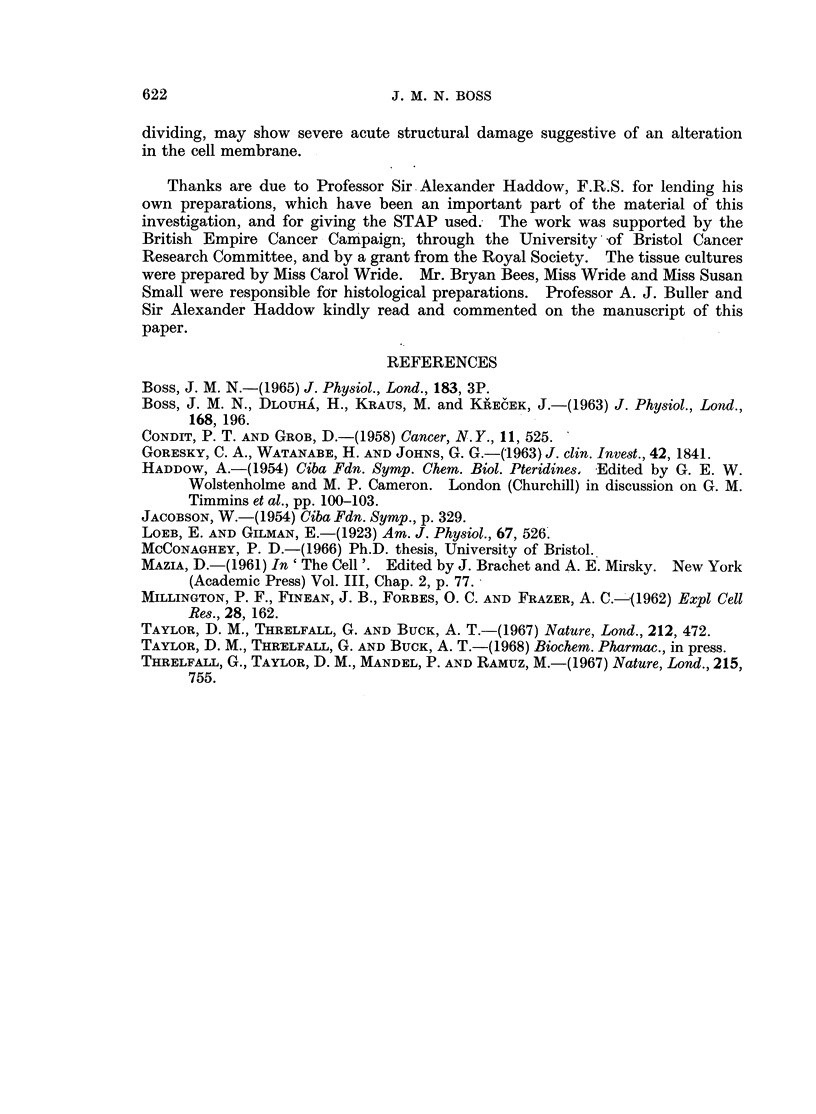# The provocation of mitosis by 6-styryl-2,4,5-triamino-pyrimidine.

**DOI:** 10.1038/bjc.1968.73

**Published:** 1968-09

**Authors:** J. M. Boss


### THE PROVOCATION OF MITOSIS BY 6-STYRYL-2,4,5-TRIAMINO-PYRIMIDINE

J. M. N. BOSS

*From the Department of Physiology, University of Bristol*

Received for publication May 28, 1968

HADDOW (1954) found that 6-styryl-2,4,5-triamino-pyrimidine (STAP), a substance related chemically to pteridines as well as to pyrimidines (Fig. 1), could provoke enlargement of the kidney, with abundant mitosis, in the kidneys of adult rats, mice, guinea-pigs and hamsters.

Haddow's studies raise certain questions, which form the basis of the experiments reported in this paper. (*a*) Are non-dividing cells brought into mitosis, or is the apparent increase in mitosis due to prolongation of the process? (*b*) Is the distribution of mitosis similar to that found without experimental interference in the rapidly growing kidney of infancy? (*c*) Why is the kidney selectively affected? (*d*) Is mitosis normal in cells provoked by STAP to divide, or in the presence of the drug? (*e*) Is the mitosis a secondary effect provoked by the loss of cells irreversibly damaged by treatment? (*f*) Does STAP cause cellular changes other than those associated with cell division?

### MATERIAL AND METHODS

### *Animals*

For mitotic counts, white rats were of the Porton strain and were specific-pathogen-free. No sections of kidneys showed either nephritis or hydronephrosis.

### *Mitotic counts in rats*

Four adult male white rats (400–600 g., 9–12 months old) were injected intraperitoneally with 10 mg. STAP (Chester Beatty Research Institute: C.B. 1019; gift of Professor Sir Alexander Haddow), suspended in 3 ml. of a mixture of equal parts of 0·9 % sodium chloride solution and arachis oil, and killed 48 hours after a single dose. Two control rats were given the oil and saline without the drug. From 2 of the rats given STAP the kidneys were fixed for 6 hours in Helly's fluid, and sections stained with haematoxylin and eosin, or by the Feulgen reaction, with light green as a counterstain. From the other 2 experimental rats and from the 2 controls the kidneys were fixed for 3 hours in Carnoy's mixture (with chloroform) and stained by Feulgen and light green. A seventh rat, given no injection at all, was killed, and the jejunum fixed in Helly's fluid and stained with haematoxylin and eosin.

Tissues were fixed immediately after death and the kidneys divided longitudinally to assist penetration of the fixative. After embedding in paraffin wax, sections were cut at 6 $\mu$. All the kidneys were cut longitudinally to include all zones and to show the papillae distinctly. Feulgen preparations were used for counting mitosis. All sections were between 1 and 1·5 cm.$^2$ in area, and the included cortex contained 150–200 renal corpuscles.

One kidney from each of 3 new-born and 3 11-day-old suckling rats was fixed in Susa, and 6 $\mu$ longitudinal sections from each block stained with haematoxylin and eosin.

In counting mitotic figures, entire sections were scanned with an apochromatic oil-immersion objective (N.A. 1·3) at a total magnification of $\times$ 830. Where the microtome knife had divided a mitotic figure, the latter was included in the count, as the part remaining within the section permitted the phase of mitosis to be ascertained.

*Temporal and spatial distribution of mitosis*

Sections, already referred to, stained with haematoxylin and eosin, were used to note which parts of the nephron and which neighbouring tissues contained dividing cells, in the kidneys of sucklings and treated adults. For the study of the latter, use was also made of histological sections kindly lent by Professor Sir Alexander Haddow. Although these sections were part of the material on which an earlier report (Haddow, 1954) had been based, they had not been used for an analysis such as that presented here. These preparations were from adult rats of the Chester Beatty strain, treated with a single dose of 10 mg. STAP, as described above, and killed at the following times after injection: 24 and 48 hours, 3, 4, 6, 9 and 11 days. The kidneys had been fixed in Bouin's fixative, and the sections stained by a green triple method. For each of the times of treatment, 3, 5, 3, 2, 4, 2, 2 and 1 sections respectively were examined, in addition to the author's own preparations, already mentioned. Occasional groups of extravascular neutrophils were present at and after 48 hours, but there was no histological evidence of previous renal disease.

*Tissue culture*

Heart fibroblasts of 11-day chick embryos were grown from primary explants on cover-slips (25 $\times$ 11 mm.) in test tubes which rotated 8 times an hour. Four explants were placed in a row directly on each cover-slip, and one cover-slip in each tube. The tubes, each of which contained 2 ml. of fluid medium, were at such an angle that all parts of each cover-slip were alternately submerged and drained. The fluid medium was a mixture of horse serum (20 %), freshly prepared chick embryo extract (30 %), and Tyrode's or Gey's balanced salt solution (50 %). After 24 hours' growth, fresh medium with or without STAP was added in studies on mitosis. For experiments on damage to non-mitotic cells the vehicle was Tyrode's solution only. The numbers of cultures, the concentrations of drug and the times of treatment will be given with the results, below. For studies of mitosis, cultures were fixed at the end of an experiment in a mixture of glacial acetic acid and absolute alcohol (1 : 3) for 5 minutes, stained with Mayer's acid haemalum, and mounted whole after dehydration and clearing. For studies on non-mitotic cells, fixation was for $2\frac{1}{2}$ minutes in buffered istonic 0·5 % osmic acid followed by 10 minutes in buffered istonic 10 % formalin. Staining was in 0·1 % aqueous azure blue overnight, and tertiary butanol was used for subsequent dehydration.

### RESULTS

*Evidence for stimulation of mitosis*

As the rat is a continuously growing animal, the obvious abundance of mitosis after treatment could have been the result of delay during division, so that more mitotic figures would have been present at any one time. The figures in the first 4 lines of Table I indicate the apparent increase to be 25–80 times. If the time for a mitotic division is about half-an-hour in mammalian tissues (Mazia, 1961), a mitotic division would need to be extended to at least half a day if such prolongation were the only explanation of the results. The proportions of the 4 phases in the first 4 lines of Table I are not significantly different between experimental and control animals, and none of the cells included in these counts showed evidence of arrested division.

TABLE I.—*Differential Counts of Mitotic Figures. Numbers in Each Phase (percentages in brackets) in Entire Sections or Cultures*

| Material | Treatment | Number of slides or cultures examined* | Prophase | Metaphase | Anaphase | Telophase | Total |
|---|---|---|---|---|---|---|---|
| Adult rat kidney | STAP 10 mg. i.p. | 1 | 234(13) | 951(53) | 381(21) | 228(13) | 1794 |
| ,, (another animal) | ,, | 1 | 224(11) | 1138(56) | 398(20) | 267(13) | 2027 |
| Adult rat kidney | Vehicle i.p. (control for STAP) | 1 | 6(16) | 17(46) | 10(27) | 4(11) | 37 |
| ,, (another animal) | ,, | 1 | 5(8) | 35(52) | 10(15) | 17(25) | 67 |
| Kidneys of 3 new-born rats | None | 3 | 23(14) | 88(52) | 30(17) | 29(17) | 170 |
| Adult rat jejunum | None | 1 | 23(9) | 140(56) | 53(21) | 36(14) | 252 |
| Chick fibroblast | STAP $2\cdot1 \times 10^{-3}$ M for 1 hr. | 8 | 79(11) | 458(65·5) | 90(13) | 73(10·5) | 700 |
| ,, | Vehicle. Control for STAP | 4 | 145(15) | 411(43) | 233(24) | 170(18) | 959 |

* Where more than one preparation is indicated the corresponding counts are aggregates

As Jacobson (1954) has reported much less mitosis in untreated kidneys than is recorded here, a further observation is relevant. Sections of kidney, comparable with those used in these experiments, were obtained from 4 adult rats used as controls in an experiment of another kind. There were 30–39 mitotic figures in each section. (Hollis, unpublished).

*Comparison with infancy*

The absence from the adult kidney of the nephrogenic zone, which forms the outer renal cortex in the suckling rat, restricts the comparison of the latter with adults to cells which, if present in the young, are already highly differentiated In the adult, mitosis, neither in stimulated nor in control kidneys, was accompanied by microscopically detectable dedifferentiation or metaplasia. For example, in a mitotic cell of the proximal tubule the brush border was normal, and the only cytoplasmic changes at metaphase were a bulging of the surface towards the lumen and some disturbance of the palisade arrangement of mitochondria.

Three generalisations are possible concerning the comparison of the distribution of mitosis in infancy and under the influence of STAP. (*a*) In neither circumstance was mitosis found in either the renal corpuscles or the final, papillary stretch of the collecting ducts. (*b*) Cells of all types capable of mitosis in the suckling, and present in the adult, were capable also of mitosis in the adult kidney stimulated by STAP. (*c*) In the thin segment of Henle's loop and in cells of the blood in renal capillaries, mitosis was found in the stimulated adult but not in the suckling.

*Stimulated tissues*

All parts of the nephron were affected, with the exceptions noted. Connective tissue and blood participated in the general tendency to mitosis. The pelvic epithelium responded to the drug after only 1 day. In all slides from adult kidneys after injection of STAP, but not in controls, there was considerable dilatation of portions of the second (medullary) parts of proximal tubules and of the thick segments of Henle's loops.

The distal tubules and collecting ducts were, as is common, widely patent in controls, and no change was detected in them.

*The question of primary damage*

The greatest amount of mitosis was two days after treatment. Except in the pelvic epithelium, no abnormal interphase cells were seen before the fourth day, when mitosis was easily seen to be diminishing. Degenerate cells in the lumen of collecting ducts were seen only from the third day.

*Experiments on cell damage*

Table I shows that no one phase of mitosis was disproportionately lengthened, as far as these counts are sensitive enough to indicate. Nevertheless, the first mitosis provoked by STAP was followed within about 3 days by the appearance, in a number of sites, of abnormal cells with dense nuclear material, such as might have originated from the chromosomes of arrested metaphase. Such cells were never seen in control or developing kidneys. Clumps of these cells, as well as normal cells, dead cells and cell debris, appeared in the lumen of the collecting ducts. Occasionally, abnormal mitosis was suspected in kidneys affected by STAP. These suspected abnormalities were present in only a small proportion of mitotic figures. Some of these figures were divided by the microtome knife to leave only part in the section while others were made compact by the natural packing of tissue, so that accurate counting of abnormalities, some of which might have been diagnosable only by the malposition of one chromosome, was not possible.

For this reason the direct effect of STAP on cells was investigated with cultures of chick fibroblasts, as already described. The drug was dissolved in the balanced salt solution used to make the medium, so that its final concentration was $1{\cdot}05 \times 10^{-3}$ M. A change of medium without drug was the control procedure, and both treated and control cultures were fixed and stained, as already described, an hour after the change of fluid. The drug was put in 26 tubes containing 104 cultures, and 112 cultures in 28 tubes served as controls.

Counts of the 4 phases of mitosis in 8 treated and 4 experimental cultures are shown in the last 2 lines of Table I. The distribution of cells between the 4 phases in the treated cultures differs significantly from that expected on the distribution in the control cultures ($P < 0{\cdot}001$ by chi-squared test). Although the ratio of anaphase to prophase figures is depressed from 1·6 to 1·1, the distribution of cells among the 3 phases other than metaphase is not changed significantly ($P \simeq 0{\cdot}1$), thus, there is not the sharp drop in the proportion of cells in anaphase, with little change for prophase, that is associated with complete arrest in metaphase (Jacobson, 1954). Cells in metaphase were therefore examined further. Table II analyses in more detail the mitosis in some of the cultures reported in the last 2 lines of Table I. The distribution between the 4 classes of metaphase figure distinguished in Table II differs significantly between numbers in the treated

TABLE II.—*Abnormalities in Mitotic Figures After Treatment of Cultures of Chick Fibroblasts by STAP. Total Counts (percentages in brackets) in Entire Cultures*

| | Control | | Treated | |
|---|---|---|---|---|
| Total of mitotic figures | 704(100·0) | | 459(100·0) | |
| Prophase | | | | |
| total | 98(13·9) | | 53(11·6) | |
| Metaphase | | | | |
| total | 310(44·0) | | 305(66·4) | |
| before formation of plate | | 81(11·5) | | 42(9·2) |
| with one or more uncongressed chromosomes | | 20(2·8) | | 131(28·5) |
| normal and complete plate | | 209(29·7) | | 105(22·8) |
| clumped | | 0(0·0) | | 27(5·9) |
| Anaphase | | | | |
| total | 190(27·0) | | 50(10·8) | |
| normal | | 189(26·9) | | 46(10·0) |
| with outlying chromosomes | | 1(0·1) | | 2(0·4) |
| with bridges between daughter groups | | 0(0·0) | | 2(0·4) |
| Telophase | | | | |
| total | 106(15·1) | | 51(11·2) | |
| normal | | 106(15·1) | | 51(11·2) |
| with outlying chromosomes | | 0(0·0) | | 0(0·0) |
| with bridges between daughter nuclei | | 0(0·0) | | 0(0·0) |

cultures and the numbers expected on the basis of the control distribution ($P < 0{\cdot}001$). The increase in the proportion of metaphase figures is due to clumping and, to a greater extent, to plates with omitted chromosomes. In the cultures in which abnormalities were counted differentially, only 2 cells out of 101 in anaphase or telophase contained chromosomal material separate from the main groups. (The incidence of the same abnormality in controls was 1 in 296). The chromosomes omitted from the plate therefore appear to congress late, rather than give rise to persistent abnormality.

The figures in the last 2 lines of Table I and in Table II differ from those given in a preliminary report of this study (Boss, 1965). This is because the analysis of metaphases given here is based on 6 cultures, not 5, and because some figures in the preliminary report were inaccurately transcribed from the original records. The discrepancies can be seen to alter the results very little, and the conclusions not at all.

Of the cultures treated with STAP, fewer than a tenth were suitable for the study of mitosis. The others contained only cells that had clumped nuclei and stringy cytoplasm, such as may occur when cells are in solutions of abnormal tonicity (McConaghey, 1966).

This effect of STAP was investigated with further cultures. Treatment was with Tyrode's solution containing the substance in various concentrations, and allowed to act for 15 minutes to 4 hours. The results are set out in Table III.

TABLE III.—*Action of STAP on Non-mitotic Cells in Cultures of Chick Fibroblasts in roller Tubes. Figures Represent Number of Tubes. Each with Four Cultures*

| | | Effects | | |
|---|---|---|---|---|
| Concentration | Time of treatment (hours) | Nil | Most cells with vacuolated cytoplasm and some cells with indistinct intranuclear detail | All cells with small dense nuclei and stringy cytoplasm |
| Nil (control) | ¼ | 1 | 0 | 0 |
| | ½ | 1 | 0 | 0 |
| | 1 | 14 | 0 | 0 |
| | 2 | 4 | 0 | 0 |
| | 4 | 1 | 0 | 0 |
| $10^{-5}$ M | 1 | 11 | 0 | 0 |
| $10^{-4}$ M | ¼ | 5 | 0 | 0 |
| | ½ | 5 | 0 | 0 |
| | 1 | 5 | 14 | 1 |
| | 2 | 4 | 0 | 4 |
| | 4 | 0 | 0 | 1 |
| $10^{-3}$ M | ¼ | 0 | 0 | 1 |
| | ½ | 0 | 0 | 1 |
| | 1 | 0 | 0 | 10 |
| | 2 | 0 | 0 | 1 |
| | 4 | 0 | 0 | 1 |

For technical reasons, which are not wholly clear, the cultures reported in Table III had little mitosis. It was therefore not possible to ascertain whether any of the times of treatment or concentrations of drug would lead to the mitotic irregularities described, without direct damage to interphase cells in any culture.

Fig. 1 shows that STAP may be expected to be alkaline in solution. As this alkalinity might have caused the damage, the reaction of solutions of the compound

FIG. 1.—Structural formulae of (a) folic acid, (b) xanthopterine, and (c) styryl-triamino-pyrimidine.

in Tyrode was measured. The results were: $10^{-5}$ M pH 7·40, $10^{-4}$ M pH 7·50 and $10^{-3}$ M pH 8·05. The possibility of surface activity was excluded by comparing the surface tension of the most concentrated of these three solutions with that of water. There was a difference not greater than 5% in the rise of either solution in the same capillary tube.

### DISCUSSION

In view of the unchanged relative lengths of mitotic phases and the occurrence of few abnormal cells, a prolongation of the mitotic process is so improbable that it must be concluded that STAP truly provokes the onset of mitosis.

From ealier reports it might be supposed that untreated adult rats had virtually no mitosis in the kidney (Jacobson, 1954). The counts in control animals in the experiments reported here differ little from those obtained in our laboratory in other studies, and do not therefore seem to be due to the arachis oil or saline. The matter can be simplified by the use of hamsters, as these respond to the drug (Haddow, 1954) but do not grow throughout their lives. Haddow is not explicit whether the hamsters used were past the first growing period of life.

The experiments reported here indicate something of the complexity of the relationship of mitosis to differentiation especially if mitosis in development (see also Boss *et al.*, 1963) is considered together with artificial stimulation in adults.

In the new-born animal, mitosis in the renal cortex is predominantly in the undifferentiated nephrongenic zone. As the cells of this zone differentiate, and there ceases to be a stock of undifferentiated cells, mitosis becomes abundant in more differentiated tubules and, in the untreated adult, fully differentiated tubule cells are found in division.

These experiments show 4 degrees of susceptibility to mitosis among differentiated cells of the nephron. First, there are cells which are in division in the developing kidney, the normal adult kidney of the rat, and the kidney provoked by STAP; such are the cells of the proximal convuluted tubule. Seondly, there are cells, such as those of the thick segment of Henle's loop, which divide in the suckling and in the treated adult, but not in untreated adults. A third group divide only in the experimental animals; the cells of the thin segments of Henle's loops are of such a kind. Lastly, there are cells, such as those of the terminal stretches of collecting ducts, which divide under none of the three conditions. Whether any kidney cells are incapable of mitosis under all circumstances cannot be inferred from these studies.

It would be reasonable to suppose that the kidney's specific susceptibility to mitosis under the influence of STAP is due to the concentration of the drug during the formation of urine. Let it be supposed that STAP is, like folic acid, with which it has much of its structure in common (Fig. 1), only partially absorbed from the tubules (Condit and Grob, 1958), or absorbed initially into tubular cells and transmitted by them only when they are saturated (Goresky *et al.*, 1963). Then a high concentration of the compound would develop in the tubular fluid bathing the cells or in the cells themselves. However, such an explanation does not take into account the provocation of mitosis in blood and other connective tissue.

On the other hand, if the concentration of the drug were due to the mechanism causing normal hypertonicity of medullary tissues in the concentrating kidney, the effect of STAP on non-epithelial structures would be explained for all the tissues of the medulla. However, the mitosis in the cortex, in both epithelium and connective tissue, would not be accounted for. Even if STAP is concentrated by both a specific limitation of tubular reabsorption and by the mechanism causing medullary hypertonicity, there is still no explanation for mitosis in cortical connective tissue if the renal specificity of STAP depends only on concentration. Taylor *et al.* (1968) have shown that STAP may block tubules, and that uretric ligation leads to acute ipsilateral renal hypertrophy. They infer a non-specific effect of obstruction. In the present studies, however, mitosis has been provoked in the renal pelvis, downstream from any tubular obstruction.

The sequence of mitosis, cellular abnormality and separation of cells into the tubular lumen begins with the provocation of cell division. Later, cells are rejected from epithelia and appear in masses in the lumen of collecting ducts. Examination of cells in the epithelium and in the lumen suggests that some of the cells which are shed might be abnormal products of mitosis arrested at metaphase, or could be derived from normal cells which have died; they may have been rejected in the restoration of the previous size of the organ.

Apart from the abnormal mitosis observed in the kidney, there is little in common between the renal changes described here and the effects of STAP on cell cultures. Indeed, it is probable that the concentrations used *in vitro* were much higher than those achieved *in vivo*. That a substance provoking cell division should also interefere with the mitotic process is not surprising if one considers the chemical relationship of STAP to pteridines (Fig. 1) and the consequent possibility that it may act as an anti-fol substance. Pteridines themselves are not only necessary for mitosis (Jacobson, 1954), but folic acid can stimulate renal growth (Taylor *et al.*, 1967; Threlfall *et al.*, 1967), and investigations in our laboratory show that this growth involves histologically demonstrable mitosis (Hollis, unpublished).

In the description of the immediate changes in non-dividing cells caused by STAP in tissue culture, comparison was made with osmotic damage. If STAP, a substance affecting the physiology of mitosis, also has a direct action of cell surfaces, it may be compared with aminopterine (Millington *et al.*, 1962). This direct damage can hardly be due to the alkalinity of the solutions used, as it occurred at pH 7·5. Loeb and Gilman (1923) found that the changes in Limulus amoebocytes exposed to alkali were a lifting of the membrane, and bursting; STAP caused shrivelling in the present studies.

### SUMMARY

1. A single intraperitoneal dose of 6-styryl-2,4,5-triamino-pyrimidine (STAP) causes, in the kidney of the adult rat, an increase of mitosis. The substance provokes division and does not merely prolong it. The mitosis is not secondary to cell damage visible by light microscopy.

2. The distribution of mitosis in the kidneys of untreated adults and sucklings is compared with that due to the drug.

3. The competence of the kidney, in particular, to react to the drug seems not to be explicable entirely in terms of renal concentration.

4. STAP causes metaphase abnormalities, especially delayed congression of chromosomes, in cultures of embryonic chick fibroblasts. Such cells, when not dividing, may show severe acute structural damage suggestive of an alteration in the cell membrane.

Thanks are due to Professor Sir Alexander Haddow, F.R.S. for lending his own preparations, which have been an important part of the material of this investigation, and for giving the STAP used. The work was supported by the British Empire Cancer Campaign, through the University of Bristol Cancer Research Committee, and by a grant from the Royal Society. The tissue cultures were prepared by Miss Carol Wride. Mr. Bryan Bees, Miss Wride and Miss Susan Small were responsible for histological preparations. Professor A. J. Buller and Sir Alexander Haddow kindly read and commented on the manuscript of this paper.

### REFERENCES

Boss, J. M. N.—(1965) *J. Physiol., Lond.*, **183**, 3P.

Boss, J. M. N., Dlouhá, H., Kraus, M. and Křeček, J.—(1963) *J. Physiol., Lond.*, **168**, 196.

Condit, P. T. and Grob, D.—(1958) *Cancer, N.Y.*, **11**, 525.

Goresky, C. A., Watanabe, H. and Johns, G. G.—(1963) *J. clin. Invest.*, **42**, 1841.

Haddow, A.—(1954) *Ciba Fdn. Symp. Chem. Biol. Pteridines.* Edited by G. E. W. Wolstenholme and M. P. Cameron. London (Churchill) in discussion on G. M. Timmins *et al.*, pp. 100–103.

Jacobson, W.—(1954) *Ciba Fdn. Symp.*, p. 329.

Loeb, E. and Gilman, E.—(1923) *Am. J. Physiol.*, **67**, 526.

McConaghey, P. D.—(1966) Ph.D. thesis, University of Bristol.

Mazia, D.—(1961) *In* ' The Cell '. Edited by J. Brachet and A. E. Mirsky. New York (Academic Press) Vol. III, Chap. 2, p. 77.

Millington, P. F., Finean, J. B., Forbes, O. C. and Frazer, A. C.—(1962) *Expl Cell Res.*, **28**, 162.

Taylor, D. M., Threlfall, G. and Buck, A. T.—(1967) *Nature, Lond.*, **212**, 472.

Taylor, D. M., Threlfall, G. and Buck, A. T.—(1968) *Biochem. Pharmac.*, in press.

Threlfall, G., Taylor, D. M., Mandel, P. and Ramuz, M.—(1967) *Nature, Lond.*, **215**, 755.